# Linking Emotional Intelligence to Mental Health in Chinese High School Teachers: The Mediating Role of Perceived Organizational Justice

**DOI:** 10.3389/fpsyg.2021.810727

**Published:** 2022-01-06

**Authors:** Sha Shen, Tianqi Tang, Hong Shu, Saidi Wang, Xiangli Guan, Xiangdong Yan, Yanli Wang, Yun Qi, Rui Feng

**Affiliations:** ^1^Key Laboratory of China’s Ethnic Languages and Information Technology of Ministry of Education, Northwest Minzu University, Lanzhou, China; ^2^College of Educational Science and Technology, Northwest Minzu University, Lanzhou, China; ^3^Gansu 24 Refractive New Media Technology Co., Ltd., Lanzhou, China; ^4^Teacher Education College, Yu Xi Normal University, Yu Xi, China; ^5^Shanghai Hui Ye (Lan Zhou) Law Office, Lanzhou, China; ^6^School of Journalism and Media, Yangzhou University, Yangzhou, China

**Keywords:** emotional intelligence, mental health, perceived organizational justice, high school teachers, teachers’ health

## Abstract

Compare with other professions, teachers are reported to have a higher risk of poor mental health. This study examined the relationships between emotional intelligence, perceived organizational justice, and mental health among Chinese high school teachers. Three hundred and eighty-one high school teachers, with their age range between 21 and 50 years, were administered the Emotional Intelligence Scale, Perceived Organizational Justice Scale, and Mental Health Scale. The result found that emotional intelligence and perceived organizational justice directly influence the mental health of high school teachers. In addition, perceived organizational justice mediated the association between emotional intelligence and mental health. Moreover, the present study analyzes the different role of subtypes of perceived organizational justice on the relationships between emotional intelligence and mental health, and the results showed that the mediating effects of perceived distributive justice and interactive justice on emotional intelligence and mental health are not significant, only the perceived procedural justice mediated the relationships between emotional intelligence and teachers’ mental health. The results are discussed in a conceptual context.

## Introduction

With the strict requirements of the society on the professional development of teachers, contemporary teachers face more psychological pressure than before (Ali et al., 2021; Hollett et al., 2021). In addition, teachers have to assume multiple roles (Naylor, 2001), which makes them more prone to psychological problems (Narayanappa et al., 2016; Yang et al., 2019). Thus, compared with other professions, teachers are reported to have a higher risk of poor mental health (Stansfeld et al., 2011; Kidger et al., 2016). Riches of studies indicated that mental health is important for teachers (e.g., Narayanappa et al., 2016; Navinés et al., 2016). The mental health of teachers not only has a strong impact on teaching skills that in turn can affect the academic achievement of students (Yang et al., 2019), but also influences developing and establishing good relationships with students (Jennings and Greenberg, 2009), which is associated with worse mental health of students (Kidger et al., 2012; Plenty et al., 2014). The mental health of high school teachers is more important, because a number of mental problems develop in adolescence (e.g., Kim-Cohen et al., 2003; Joinson et al., 2017) and the mental health of teachers is closely related to that of students (Klusmann et al., 2016; Harding et al., 2019). Therefore, more research is needed to further clarify the factors and mechanisms that affect the mental health of high school teachers. This question is important because identifying the determinants and mechanisms of mental health among high school teachers is a critical early step for implementing interventions.

Teachers often face negative emotions in their interactions with students, family members, and colleagues (Travers, 2017); it is reasonable that emotional intelligence is regarded as one of the factors influencing mental health of teachers (Antinienė and Lekavičienė, 2017). Emotional intelligence is defined as “the extent to which a person processes emotional information adequately” (Mayer et al., 2000, 2016). Ample evidence showed that emotional intelligence is associated with classic indicators of mental health significantly (e.g., Cejudo, 2016; Singh and Bhardwaj, 2016). For example, Lea et al. (2019) used meta-analyses method to investigate the relationships between emotional intelligence and mental health, and found that higher emotional intelligence is associated with lower levels of depression, anxiety, and psychological distress and less likely to suicide (García-Sancho et al., 2014). A follow-up study showed that the students who with high emotional intelligence had fewer symptoms of depression and anxiety over time (Antinienė and Lekavičienė, 2017), and an international research has confirmed that high emotional intelligence can significantly improve the level of mental health (Kaur, 2019).

Although the previous studies indicated that emotional intelligence is related to mental health, the specific mechanisms involved in the association remain unclear. For example, mental health may be influenced by emotional intelligence through perceived organizational justice (POJ). POJ refers to employees’ perception of fairness in their organizations (Eib et al., 2017), when individuals realize that a decision or process is unfair, they may develop a negative attitude toward work, reduce motivation to work (Skarlicki and Folger, 1997; Cordes, 2010). According to the emotional intelligence theory proposed by Salovey and Mayer (1990), teachers with higher emotional intelligence can better manage and use their emotions and those of others in school, can communicate well with other colleagues and leaders in school, and promote an understanding of organization-related factors and equity factors in organizations. On the contrary, individuals with relatively low emotional intelligence have difficulty in controlling their emotions and are prone to organizational injustice. In line with this model, POJ has been shown to be associated with emotional intelligence (Zeidner et al., 2004; Ouyang et al., 2015). For instance, Ouyang et al. (2015) found that there is a significant correlation between POJ and emotional intelligence. Specifically, higher level of emotional intelligence is associated with higher level of POJ. In addition, researchers have been investigating new psychosocial stressors, such as organizational justice in the context of mental health (Ferrie et al., 2006). Studies have shown that POJ is not only a major determinant of behavior and attitude outcomes, but also an important psychosocial predictor of individual health (Weiss et al., 1999). Previous studies have shown that there is a significant association between POJ and mental health. Specifically, lower level of POJ is associated with worse mental health (e.g., Hayashi et al., 2011; Rai, 2015). So, does POJ mediates the relationships between emotional intelligence and mental health?

In addition, based on the previous literature, POJ including three basic aspects: perceived distributive justice, perceived procedural justice, and perceived interactional justice. Perceived distributive justice refers to perception of fairness in rewards or resources that are allocated, perceived procedural justice represents an individual’s view of the fairness of the process by which the administration makes decisions, and perceived interactional justice refers to the fairness of interpersonal treatment perceived by individuals in the process of decision implementation (Colquitt and Greenberg, 2003, p. 159). These subtypes of POJ are conceptually distinct (Erdogan, 2002; Ambrose and Arnaud, 2005) and have different influencing factors and different influences on individuals (Colquitt et al., 2001; Erdogan, 2002). To the authors’ knowledge, no research has investigated the role of different subtypes of POJ in the relationships between emotional intelligence and mental health among teachers. So, does perceived distributive justice, perceived procedural justice, and perceived interactional justice play different roles in the relationships between emotional intelligence and mental health among high school teachers?

The present study investigates whether the relationships between emotional intelligence and mental health influenced by POJ among high school teachers. More important, the present study investigates whether the subtypes of POJ play different roles in the relationships between emotional intelligence and mental health. Based on previous studies, the present study hypotheses that the POJ mediates the relationships between emotional intelligence and mental health, and perceived distributive justice, perceived procedural justice, and perceived interactional justice play different roles in the relationships between emotional intelligence and mental health among high school teachers.

## Materials and Methods

### Participants

The participants were 401 high school teachers from three high schools in northern city using utilizing convenience sampling technique. The age range was 21–50 (*M* = 35.95, SD = 7.78). Any high school teacher who did not participate in a similar study can volunteer to participate. After excluding 20 incomplete questionnaires (5%), a total of 381 responses of participants (182 males and 199 females) were used in the present study. Other demographic information is presented in Table 1.

**Table 1:** Socio-demographic characteristics of the participants (*n* = 381).

	Groups	Frequency (%)
**Gender**		
Female	182	47.8
Male	199	52.2
**Age**		
<35	178	46.72
36–45	154	40.42
46–50	49	12.86
**Education ground**		
College degree	44	11.55
Bachelor degree	327	85.83
Graduate degree	10	2.62
**Grade of teaching**		
Senior one	124	39.90
Senior two	132	37.27
Senior three	125	22.83

At the end of the experiment, all participants received a gift worth 30 RMB. The research proposal was approved by the local academic committee.

### Measures

#### Emotional Intelligence Scale

The Emotional Intelligence Scale (EIS) consists of 16 items (Law et al., 2004) and some examples of items including “I have a good understanding of my own emotions” and “I always knew if I was happy or not.” Each item is answered on a seven-point scale (1 = strongly disagree and 7 = strongly agree). The scores used in the present study were calculated by summing all the scores of each item, and higher scores indicated higher emotional intelligence. The Chinese version of EIS has satisfactory reliability and validity (e.g., Hu et al., 2016; Geng, 2018). In the present study, the EIS’s Cronbach alpha coefficient was 0.93.

#### Perceived Organizational Justice

The scale of POJ consists of 20 items (Niehoff and Moorman, 1993) and some examples of items including “I feel I am being rewarded fairly considering the responsibilities” and “My work is arranged fairly.” Each item is answered on a five-point scale (1 = strongly disagree and 5 = strongly agree). The scores used in the present study were calculated by summing all the scores of each item, and higher scores indicated experiencing higher level of organizational justice. This scale is frequently used in China and has good validity and reliability (Ouyang et al., 2015). In the present study, the POJ’s Cronbach alpha coefficient was 0.91.

#### Mental Health Scale

The scale of mental health scale (MHS) consists of 30 items (Cheng et al., 1990) and some examples of items including “how much sleep about worry?” and “been feeling giddy?.” Each item is answered on a five-point scale (5 = strongly disagree and 1 = strongly agree). The scores used in the present study were calculated by summing all the scores of each item, and higher scores indicated experiencing better mental health. This scale is frequently used in China and has good validity and reliability (Cheng et al., 1990). In the present study, the MHS’s Cronbach alpha coefficient was 0.90.

### Procedure

We contacted principles of three high schools in the northern city of China to describe the purpose of the present study. They approved the study and allowed questionnaires to be sent to teachers. 401 teachers volunteered to take part in the survey. All questionnaires were completed in the school office after the teachers had completed informed consent.

### Ethics

This study is based on the Declaration of Helsinki and its subsequent amendments. The study protocol was approved by the Ethics Committee of the Northwest Minzu University.

### Analytical Strategy

SPSS 25.0 was used to analyze the data. First, we established the relationships between emotional intelligence, mental health, and POJ by the correlation analysis. Then, we conducted the two-step procedure proposed by Anderson and Gerbing (1988) and used SPSS macro PROCESS program to examine the mediating effects.

## Results

### Descriptive Statistics and Correlations for Emotional Intelligence, POJ, and Mental Health

The descriptive statistics and the correlation test were conducted for emotional intelligence, POJ, and mental health and the results were shown in Table 2. The results showed that emotional intelligence, POJ, and mental health were significantly correlated (*p* < 0.01).

**Table 2:** Descriptive statistics and correlations for all variables (*n* = 381).

Variable	*M* (SD)	1	2	3
Emotional intelligence	56.15 (12.22)	1		
Perceived organizational justice	52.9 (11.24)	0.21^**^	1	
Mental health	71.86 (8.76)	0.46^**^	0.45^**^	1

** *p* < 0.01.

### The Analysis of the Mediating Role of POJ Between Emotional Intelligence and Mental Health

In order to analyze the effect of emotional intelligence on mental health and the role of POJ, SPSS macro PROCESS program was used to test the mediating effect. The mediation model testing needs to estimate the parameters of two regression equations: First, the direct effect of independent variable (emotional intelligence) on dependent variable (mental health); Second, the mediating effect (indirect effect) of POJ on the independent variable (emotional intelligence) and the dependent variable (mental health).

**Figure 1:**
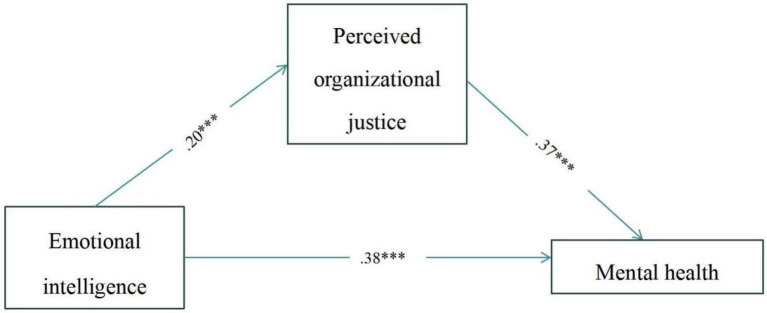
The model of emotional intelligence, perceived organizational justice (POJ), and mental health.

If the model meets the following conditions, the mediation effect exists as: (1) Emotional intelligence has a significant effect on mental health; (2) Emotional intelligence has a significant effect on POJ; and (3) POJ has a significant effect on mental health. The results of regression analysis are shown in Table 3. The results showed that emotional intelligence predicted mental health significantly (*β* = 0.46, *t* = 10.03, *p* < 0.001). In addition, emotional intelligence predicted POJ significantly (*β* = 0.20, *t* = 4.07, *p* < 0.001), while POJ predicted mental health significantly (*β* = 0.37, *t* = 8.69, *p* < 0.001). As shown in Figure 1, POJ plays a mediating role on the relationships between emotional intelligence and mental health.

**Table 3:** The test of mediation effect (*n* = 381).

Variable	Model 1			Model 2		
	Mental health			Mental health		
	Effect of value	SE	*t*	Effect of value	SE	*t*
Emotional intelligence	0.46	0.03	10.03^***^	0.38	0.03	8.97^***^
Perceived organizational justice				0.37	0.033	8.69^***^
*R^2^*	0.21			0.34		
*F*	12.95^***^			10.97^***^		

*** *p* < 0.001.

In order to obtain reliable results of mediating effect, the mediation effect was further tested using the non-parametric percentage Bootstrap which corrected by deviation. If the confidence interval of 95% Bootstrap does not contain 0, the mediating effect is significant. The results are shown in Table 4. The direct effect value of emotional intelligence on mental health is 0.27, accounting for 82% of the total effect. The 95% interval is [0.21, 0.33], indicating that the direct effect is significant. The indirect effect of POJ on emotional intelligence and mental health is 0.05, accounting for 18% of the total effect, with a 95% interval of [0.26, 0.88], indicating a significant mediating effect.

**Table 4:** Bootstrap analysis of significance test of mediation effect (*n* = 381).

The path	Effect of value	Effect of the amount (%)	Bootstrap 95% confidence interval down line	Bootstrap 95% confidence interval upper line
Direct effect (A → C)	0.27	82%	0.21	0.33
Indirect effect (A → B → C)	0.05	18	0.26	0.88
Total effect	0.33	100	0.26	0.39

A, emotional intelligence; B, perceived organizational justice; and C, mental health.

### The Analysis of the Different Role of the Subtypes of POJ Between Emotional Intelligence and Mental Health

**Figure 2:**
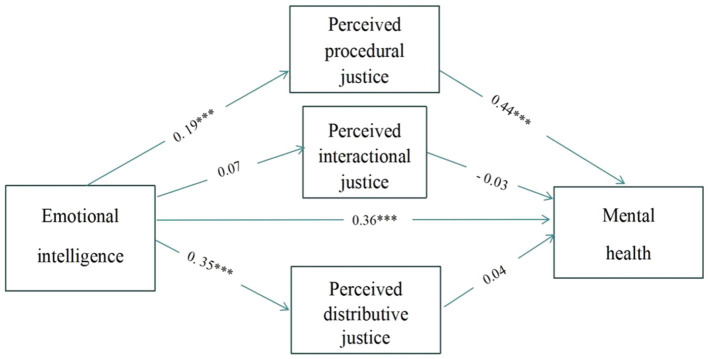
The model of emotional intelligence, different of subtypes of perceived organizational justice and mental health.

In order to analyze the effect of emotional intelligence on mental health and the different role of subtypes of POJ, SPSS macro PROCESS program was used to test the mediating effect. The three dimensions of POJ were conducted as multiple mediating variables. As shown in Figure 2, the results showed that the mediating effects of perceived distributive justice and perceived interactive justice on emotional intelligence and mental health are not significant, only the mediating effect of perceived procedural justice on emotional intelligence and mental health was found [*β* = 0.44, *p* < 0.001, SE = 0.03, *t* = 9.16, 95% CI = (0.71, 1.16)], and the mediating effect accounted for 21.2% of the total effect.

## Discussion

The main aim of the present study was to examine the mediating effect of POJ on emotional intelligence and mental health among Chinese high school teachers. The results of this study showed that POJ mediates the relationships between emotional intelligence and mental health. More importantly, the present study also investigated the different role of subtypes of POJ and found only the perceived procedural justice mediates the relationships between emotional intelligence and mental health of teachers.

The results of the present study expand the reports of potential causes of teacher’s mental health from other studies (Yang et al., 2019; Halladay et al., 2020; Ain et al., 2021) and also reveal the determinants and mechanisms between emotional intelligence and teachers’ mental health. As a result of the analysis which was conducted for examining the direct role of emotional intelligence on mental health was found significant prediction. Riches of previous studies also found out that emotional intelligence had a significant relationship with mental health (e.g., García-Sancho et al., 2014; Lea et al., 2019). The present study found a direct predict effect of emotional intelligence on mental health. This is in line with the emotional intelligence theory (Salovey and Mayer, 1990), according to which, emotional intelligence is an important kind of ability to regulate and understand own and others’ emotions (Mayer et al., 1990). Teachers often face negative emotions (Travers, 2017), and teachers with high emotional intelligence are more likely to monitor their own feelings and emotions, and are good at using this information to guide their own thinking and actions. As a result, they are more likely to be in good mental health.

In addition, the present study emphasizes that POJ accounted for the relationships between emotional intelligence and mental health among high school teachers. This is in line with the emotional intelligence theory (Salovey and Mayer, 1990), according to which, individuals with relatively low emotional intelligence have difficulty in controlling their emotions and are prone to organizational injustice. In addition, this is in accordance with job demands-resources model (Demerouti et al., 2001). According to the job demands-resources model, the higher an individual’s POJ is, the more resources they feel they have, which will weaken the impact of job requirements and alleviate individual’s mental health, which implies that a high sense of organizational justice associated with better mental health.

More importantly, the present study found only the perceived procedural justice mediates the relationships between emotional intelligence and teachers’ mental health. The results in line with conservation of resources theory (COR). According to COR theory, individuals strive to conserve and utilize psychological resources, such as energy and personal identity (Halbesleben et al., 2014). The theory holds that the resource loss is more critical than the resource gain (Hobfoll, 1989). The perception of procedural justice mainly comes from whether the allocation process of resources is fair or not. If individuals perceive that the allocation process is unfair to themselves, they will perceive that their resources are reduced. Such perception of reduced resources will exert pressure on individuals and lead them to adopt some defensive strategies to avoid resource loss in future (Halbesleben et al., 2014), thus leading to a decline of their mental health.

There are some limitations in this study. First, this study is cross-sectional, no causal conclusions can be drawn. More longitudinal studies should be conducted in the future to verify the causal relationship among these variables. Second, this study was conducted among Chinese teachers, the results obtained may not be generalized to relevant populations in other countries. Third, the participants were mostly from northern cities of China, the representativeness of the sample should be expanded in future studies.

Despite its limitations, this study is the first attempt to investigate the underlying mechanism of POJ between emotional intelligence and mental health among Chinese high school teachers. The results indicate that emotional intelligence influences mental health by POJ, and only the perceived procedural justice mediates the relationships between emotional intelligence and teachers’ mental health. These results may provide valuable information for the intervention design aimed at reducing the factors affecting teachers’ mental health. On the one hand, improving teachers’ emotional intelligence through training can influence the relationships between emotional intelligence and mental health. On the other hand, for teachers with low emotional intelligence, the relationships between emotional intelligence and mental health can be affected by improving perceived procedural justice.

## Data Availability Statement

The raw data supporting the conclusions of this article will be made available by the authors, without undue reservation.

## Ethics Statement

The studies involving human participants were reviewed and approved by the Ethics Committee of the College of Educational Science and Technology of Northwest Minzu University. The patients/participants provided their written informed consent to participate in this study.

## Author Contributions

SS, XG, RF, and SW proposed the original thoughts. TT, XY, YW, YQ, and HS collected the data. All authors contributed to the article and approved the submitted version.

## Funding

This work was supported by the Key Laboratory of Ethnic Languages and Information Technology of Ministry of Education of China, Northwest Minzu University, Lanzhou, Gansu, China (KFKT202013, KFKT202016, KFKT202012, and 1001161310). The Young Doctor Foundation of Higher Education in Gansu Province “Research on the educational effect mechanism of the socialist core value ‘unity of knowing and doing’ of college students for nationalities in the new era” (no. 2021QB-071). The 14th five-year planning project of Education science in Gansu Province “A study on the core behavior of patriotism of children in the new era” (no.: GS[2021]GHB1836). Fundamental Research Funds for the Central Universities “Research on influencing factors of online education in post-epidemic era” (no.: 31920210125).

## Conflict of Interest

SW was employed by Gansu 24 refractive new media technology Co., Ltd. XY was employed by Shanghai Hui Ye (Lan Zhou) Law Office.

The remaining authors declare that the research was conducted in the absence of any commercial or financial relationships that could be construed as a potential conflict of interest.

## Publisher’s Note

All claims expressed in this article are solely those of the authors and do not necessarily represent those of their affiliated organizations, or those of the publisher, the editors and the reviewers. Any product that may be evaluated in this article, or claim that may be made by its manufacturer, is not guaranteed or endorsed by the publisher.
